# Sustained High Level of Serum VEGF at Convalescent Stage Contributes to the Renal Recovery after HTNV Infection in Patients with Hemorrhagic Fever with Renal Syndrome

**DOI:** 10.1155/2012/812386

**Published:** 2012-10-14

**Authors:** Ying Ma, Bei Liu, Bin Yuan, Jiuping Wang, Haitao Yu, Yun Zhang, Zhuwei Xu, Yusi Zhang, Jing Yi, Chunmei Zhang, Xingchun Zhou, Angang Yang, Ran Zhuang, Boquan Jin

**Affiliations:** ^1^Department of Immunology, The Fourth Military Medical University, 169 Changle West Road, Xi'an 710032, China; ^2^Department of Infectious Diseases, Tangdu Hospital, The Fourth Military Medical University, Xi'an 710032, China

## Abstract

To investigate the role of vascular endothelial growth factor (VEGF) in the increased permeability of vascular endothelial cells after Hantaan virus (HTNV) infection in humans, the concentration of VEGF in serum from HTNV infected patients was quantified with sandwich ELISA. Generally, the level of serum VEGF in patients was elevated to 607.0 (542.2–671.9) pg/mL, which was dramatically higher compared with healthy controls (*P* < 0.001). There was a rapid increase of the serum VEGF level in all patients from the fever onset to oliguric stage, at which the serum creatinine reached the peak level of the disease, indicating that VEGF may be involved in the pathogenesis of renal hyper-permeability. Moreover, the serum VEGF level at convalescent stage was positively correlated with the degree of the disease severity. The sustained high level of serum VEGF at convalescence was observed in critical HFRS patients, suggesting that VEGF would probably contribute to the renal recovery after the virus clearance. Taken together, our results suggested that the VEGF would be involved in the pathogenesis of renal dysfunction at the oliguric stage after HTNV infection, but may function as a recovery factor during the convalescence to help the body self-repair of the renal injury.

## 1. Introduction

Hantaan virus (HTNV), the prototype of the Hantavirus genus, is a rodent-borne pathogen which could cause severe hemorrhagic fever with renal syndrome (HFRS) in human with a mortality rate up to 10% mainly in Asia [1, 2]. Plasma leakage is a hallmark of the syndrome, which is characterized by acute thrombocytopenia, the loss of vascular integrity, and enhanced vascular permeability, leading to hemorrhage in patient days to weeks after infection [3, 4]. Renal is the major organ that would be pathologically damaged during HFRS with the most prominent finding of acute tubulointerstitial nephritis with infiltration of inflammatory cells [5, 6]. However, the clinical course of HFRS is self-limited and the renal injury of the patients could gradually recover after discharge from the hospital.

Acute renal failure accompanied with Hantavirus infection is closely linked to the endothelial damage by various cytokines and other humoral factors [7]. All Hantaviruses predominantly infect endothelial cells. Hantaviruses replicate in the cytoplasm and mature by budding into the lumen of the Golgi complex, where their surface glycoproteins are trafficked, and exit cells by an abnormal secretory process [8]. However, they do not lyse the endothelial cells [3, 9–11]. The absence of a cytopathic effect has also been reported in *in vitro *studies of Hantavirus infection of human primary endothelial cells [10, 12]. Vascular permeability occurs with a relative lack of cytopathic effect, suggesting that there may be a transient change of the normal function of endothelial cells, which play a primary role in maintaining the fluid barrier of the vasculature integrity [13–15]. 

It has been believed that the induction of an uncontrolled immune response to the Hantavirus infection, rather than the viral infection, is the cause of the microvascular leakage and, ultimately, development of the HFRS or Hantavirus pulmonary syndrome (HPS). Some studies have also indicated that vascular barrier functions are affected both directly by the virus and indirectly through the increased synthesis and release of proinflammatory cytokines. Indeed, T cells and cytokines have been suggested to be involved in permeability, but their contribution to HFRS or HPS diseases remains undefined [9, 16, 17]. Vascular endothelial growth factor (VEGF), the most potent permeability-enhancing cytokine, plays a role in angiogenesis, is involved in capillary permeability, stimulates endothelial cell differentiation, proliferation, and migration, and supports vascular survival by preventing endothelial apoptosis [14, 18]. The human VEGF gene family consists of five members of VEGF A-E, among which VEGF-A is also commonly referred to as VEGF. It was reported that VEGF could exert its physiologic functions via binding to its homologous membrane tyrosine kinase receptor, VEGFR2, which is expressly restricted on endothelial cells [19, 20]. In the study of Dengue virus, an elevated plasma level of VEGF-A associated with a decrease in its soluble receptor, VEGFR2 were observed in patients with Dengue hemorrhagic fever [21, 22]. Moreover, it has also been well accepted that *β*3 integrins serve as particularly Hantavirus receptors and are highly expressed on the surface of endothelial cells. Interestingly, the *β*3 integrins and VEGFR2 can form a functional complex and interact with each other [23]. Thus, the interaction of pathogenic Hantaviruses with *β*3 integrins in concert with VEGF might be important determinant for pathogenesis caused by Hantaviruses *in vivo*. Moreover, many studies suggested that VEGF would be involved in recovery from various forms of renal injury, such as the acute kidney injury, glomerulonephritis, renal microvascular damage, and ischemia-reperfusion injury, in which VEGF could promote the recovery process of impaired vascular endothelial cells [24–27].

In this study, we detected the concentration of VEGF in the serum of HFRS patients and compared the levels of VEGF throughout the course of the illness in a large cohort of patients with different severities. The dynamic changes of serum VEGF showed that the elevation began from the fever onset, but reached the peak level at different stages of the disease depending on the disease severity. The sustained high level of serum VEGF at convalescent stage was positively correlated with the severity degree of the disease, indicating that VEGF in HFRS patients at the convalescence would probably be involved in the repairment of renal injury after HTNV infection. 

## 2. Materials and Methods

### 2.1. Study Participants

A total of 88 adults presenting to the doctors with symptoms of fever, hemorrhage, effusion, and renal abnormalities and who were prospectively identified as HFRS were enrolled in this study between 2009 and 2010 at the Department of Infectious Diseases at the Tangdu Hospital of The Fourth Military Medical University (Xi'an, China). All cases were clinically diagnosed by the detection of IgM or IgG specific antibodies to HTNV in the patients' serum specimens. According to the symptoms such as renal function, effusion, hemorrhage, and edema, the severity degree of the HFRS disease can be classified as previously described [28]: (1) mild patients were identified with mild renal failure without an obvious oliguric stage; (2) moderate for those with obvious symptoms of uremia, effusion (bulbar conjunctiva), hemorrhage (skin and mucous membrane), and renal failure with a typical oliguric stage; (3) severe patients with severe uremia, effusion (bulbar conjunctiva and either peritoneum or pleura), hemorrhage (skin and mucous membrane), and renal failure with oliguria (urine output, 50–500 mL/day) for ≤5 days or anuria (urine output, <50 mL/day) for ≤2 days; (4) critical ones with ≥1 of the following symptoms during severe disease: refractory shock, visceral hemorrhage, heart failure, pulmonary edema, brain edema, severe secondary infection, and severe renal failure with oliguria (urine output, 50–500 mL/day) for >5 days, anuria (urine output, <50 mL/day) for >2 days, or a blood urea nitrogen level of >42.84 mmol/L. Moreover, according to the clinical observations, this illness can be divided into five sequential stages: febrile, hypotensive, oliguric, diuretic, and convalescent. The patients who had other kidney disease, diabetes, cardiovascular disease, hematological disease, autoimmune disease, viral hepatitis, and other liver diseases were excluded.

For the present study, sixty-one healthy adult volunteers (36 men and 25 women; mean age 33.8 ± 7.6 years) without a history of HFRS-like disease were selected as normal control donors. There were no significant differences in the distribution of age and gender between HFRS patients and healthy controls.

### 2.2. Sample Collection

Blood samples were intravenously collected from the healthy donors and the HFRS patients. The serum samples were isolated from blood samples by centrifugation and cryopreserved at −20°C until use. The records of the clinical parameters were collected at the same time from the case file of each patient.

This study was approved by the Institutional Review Boards of the Tangdu Hospital and The Fourth Military Medical University. Written informed consent was obtained from both the HFRS patients and the normal healthy donors before blood collection.

### 2.3. Enzyme-Linked Immunosorbent Assay for VEGF Detection

The level of VEGF in serum was measured with sandwich enzyme-linked immunosorbent assay (ELISA) kits (Bender MedSystems) according to the manual. The system used a solid-phase polyclonal coating antibody to human VEGF-A and a biotin-conjugated antihuman VEGF-A polyclonal antibody for detection. For the assay, a 100 *μ*L serum sample for each test was used. The optical densities were determined at 450 nm. The concentration of VEGF in the tested samples was estimated from the standard curve as determined with serially diluted reconstitution VEGF-A standards. Concentrations are reported in pg/mL.

### 2.4. Statistical Analysis

Statistical analysis was performed using SPSS 16.0 (SPSS Inc) software and Prism software 5.0 (Graphpad). Continuous variables were analyzed by the Kolmogorov-Smirnov's test for normality of distribution and the Levene's test for the homogeneity of variance. The variables were reported as the mean (95% confidence interval, CI) and compared between groups with the Student's *t*-test for normally distributed variables. For the nonnormally distributed variables, the nonparametric Mann-Whitney *U*-test was used. Associations between continuous variables were analyzed by the nonparametric Spearman correlation analysis. A *P* value of less than 0.05 was considered as indicating statistical significance.

## 3. Results

### 3.1. Characteristics of the HFRS Patients

Overall, 197 serum samples were collected from the 88 HFRS patients at different phases of the disease and after discharge. According to the clinical records and diagnostic criteria, 13, 21, 26, and 28 patients were diagnosed as mild, moderate, severe, and critical HFRS, respectively. There were 32 samples at the febrile stage, 15 samples at the hypotensive stage, 34 samples at the oliguric stage, 54 samples at the diuretic stage and 25 samples at convalescence. Apart from this, 37 serum samples were collected 8 months after patients' discharge. The details of the clinical parameters detected during the hospitalization of the patients were summarized in Tables 1 and 2. There was no difference pertaining to sex and age among the four degrees of the severity in HFRS patients. It should be noted that the maximum levels of blood urea nitrogen (BUN), serum creatinine (sCr), and leukocyte counts elevated gradually in an order of disease severity (from mild to critical type), whereas the nadir count of platelets was inversely correlated with the disease severity. As related to the dynamic changes of the parameters during the course of the disease, the BUN and sCr reached the peak level at the oliguric stage. The number of leukocytes reached a maximum, whereas the platelet number and serum albumin level declined to the minimal at the hypotensive stage. Eight patients exhibited peak SCr levels of >707 *μ*mol/L, and 24 patients had severe proteinuria (+++). Interesting, the presence of atypical lymphocytes with a percent from 4% to 20% primarily at the acute stage of HFRS indicated that the lymphocytes stayed as the mitosis phase, which could be considered valuable in the diagnosis and prognosis after acute virus infection [29, 30].

### 3.2. Dramatic Elevation of the Serum VEGF Levels in HFRS Patients

The mean (95% CI) level of VEGF in the serum samples from the healthy controls was 200.9 (179.7–222.0) pg/mL, which was consistent with the previous reports [31, 32]. Compared with the healthy controls, the mean (95% CI) level of VEGF in all the serum samples from the HFRS patients irrespective of disease severity and stages was apparently elevated to 607.0 (542.2–671.9) pg/mL (*P* < 0.001 between healthy controls and HFRS patients). Considering the different severity, the mean (95% CI) level of serum VEGF was 526.0 (420.9–631.0) pg/mL in mild group, 564.1 (475.3–652.8) pg/mL in moderate group, 600.2 (451.2–749.3) pg/mL in severe group, and 690.8 (540.9–840.6) pg/mL in critical group, which were elevated in all the four groups compared with healthy controls (*P* < 0.01), but without difference between each other (*P* > 0.05). Considering the maximum value of serum VEGF in each patient during the hospitalization in different severity groups, the same results were observed (Table 1). Then, we analyzed the serum VEGF level in HFRS patients at each stage of the disease irrespective of the different severities; the mean level of serum VEGF was increased from the fever onset, reached the peak level at diuretic stage and still maintained a certain level at convalescence, and decreased to normal level when detected 8 months after discharge (Table 2).

### 3.3. The Dynamic Changes in Serum VEGF Level in Different Severity Groups in HFRS Patients

**Figure 1:**
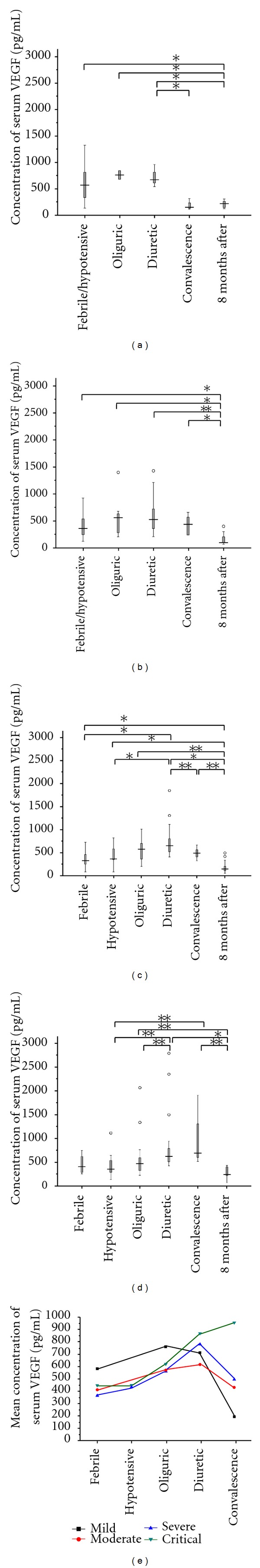
Compare the concentration of serum VEGF among different stages and 8 months after discharge in each severity group of HFRS patients. (a) Mild patients, (b) moderate patients, (c) severe patients, and (d) critical patients were investigated. The level of serum VEGF in different severity groups was generally elevated from onset of fever and then gradually elevated. It could recover to the normal level 8 months after discharge irrespective of the severity of the patients. (e) The dynamic changes in the mean level of serum VEGF at each stage of HFRS in four different severity groups were investigated. Mann-Whitney *U*-test or Student's *t*-test was used for a two-group comparison. *P < 0.05, **P < 0.01.

In general, the serum VEGF levels in HFRS patients at different clinical stages in four groups were almost elevated compared with those in healthy controls. It decreased to the normal level when detected 8 months after discharge from hospital in all the patients (*P* > 0.05 compared with healthy controls) (Figures 1(a)–1(d)). The dynamic changes in different groups showed that the mean levels of serum VEGF in HFRS patients increased from the the fever onset but reached the peak level at different stages: in mild group, the peak level was performed at oliguric stage, whereas the peak level was observed at diuretic stage in moderate or severe group. Moreover, the level of serum VEGF in patients with critical severity sustained increasing during the whole course of disease and reached the peak level at convalescent stage (Figure 1(e)). Meanwhile, it should be noted that the patients with mild HFRS had a higher level of serum VEGF at febrile/hypotensive and oliguric stages than that in the other three groups, but could quickly reduce to the normal level at convalescence (*P* > 0.05 compared with healthy control) (Figure 1(e)).

### 3.4. The Level of Serum VEGF at Convalescent Stage Related to the Different Severity in HFRS Patients

**Figure 2:**
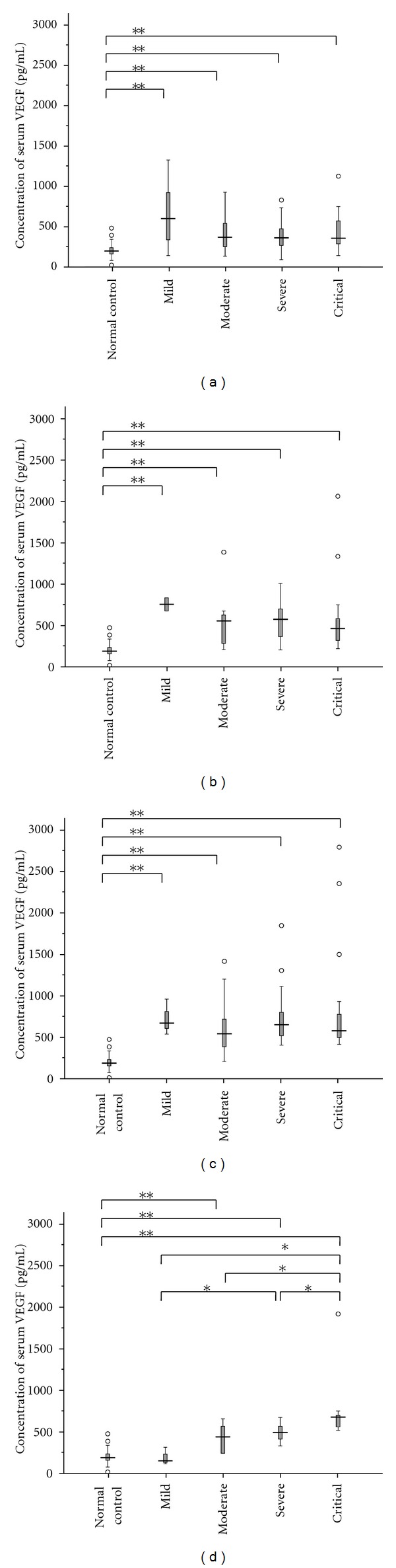
Compare the concentration of serum VEGF among four severity groups at each stage of HFRS. For (a) febrile/hypotensive stage, (b) oliguric stage, and (c) diuretic stage, the serum VEGF levels are significantly higher than those in healthy controls, but there was no difference in the serum VEGF level among the four severity groups. (d) For the convalescence stage, the level of serum VEGF was higher in the more serious group. The significant difference was performed among four groups. The Mann-Whitney *U*-test was used for a two-group comparison. *P < 0.05, **P < 0.01.

Among the four severity groups, there was no significant difference in the serum VEGF level from the onset of fever to the diuretic stage (Figures 2(a)–2(c)). However, the concentration of serum VEGF at the convalescent stage was positively correlated with the severity of the disease. Specifically, the concentration of serum VEGF was 765.7 (429.3–1102.2) pg/mL in critical patients at the convalescence stage, which was 3.96-fold compared with mild patients (193.3  (67.9–454.5) pg/mL), 1.78-fold compared with moderate patients (429.8  (198.2–661.5)  pg/mL), and 1.55 fold compared with severe patients (494.3  (398.8–598.9) pg/mL), respectively (Table 1). The level of serum VEGF at convalescence could decrease to normal level only in mild patients (*P* > 0.05). The more serious the patients got, the higher level the serum VEGF reached at the convalescent stage (Figure 2(d)).

### 3.5. The Correlation between the Level of Serum VEGF and the Clinical Parameters

**Figure 3:**
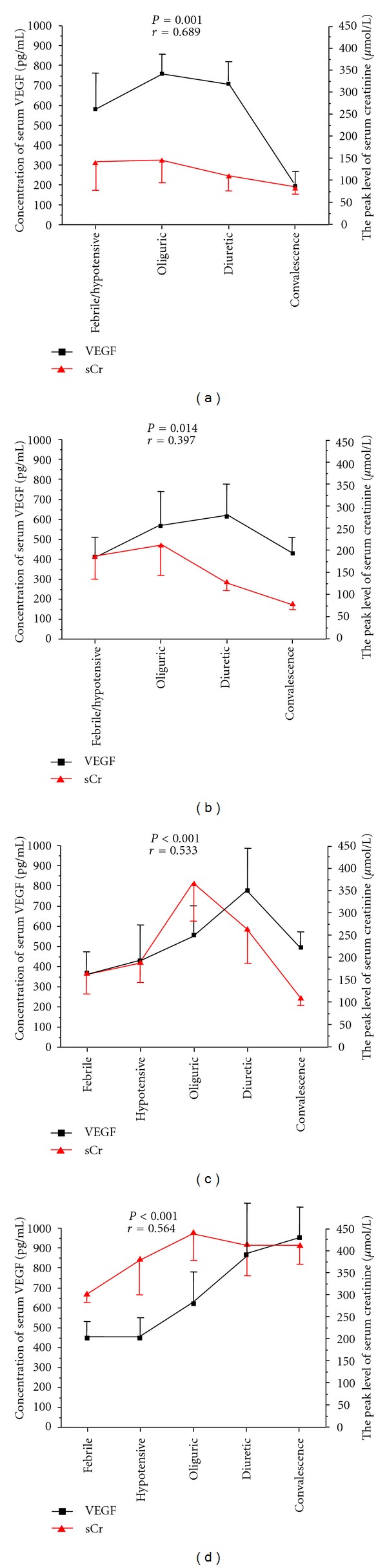
The correlation between the mean level of serum VEGF and the mean level of serum creatinine in four different severity groups at each stage of HFRS. (a) Mild patients, (b) moderate patients, (c) severe patients, and (d) critical patients were investigated. The nonparametric Spearman correlation analysis was used to evaluate the *r* and *P* values. sCr: serum creatinine.

Then, we analyzed the relationship between the serum VEGF level and the clinical parameters detected during hospitalization. The mean level of sCr significantly increased from the febrile stage, reached a peak value at the oliguric stage, and then reduced till the convalescent stage. Considering the different severity groups, the almost consistent trends between the mean level of serum VEGF level and the mean level of sCr were performed in patients with different severities (Figures 3(a)–3(d)). In mild patients, both the VEGF and sCr reached the peak level at the oliguric stage, and then gradually declined (Figure 3(a)). In patients with moderate or severe type, the sCr reached maximum at the oliguric stage, whereas the level of VEGF maintains elevated until the diuretic stage (Figures 3(b) and 3(c)). In the critical ones, the VEGF sustained increasing during the whole course of the disease, and the sCr decreased slightly from the oliguric stage (Figure 3(d)). At convalescence stage, the level of sCr could decrease to 79.63 *μ*mol/L in mild patients, 78.90 *μ*mol/L in moderate patients, and 108.80 *μ*mol/L in severe patients, whereas the sCr still sustained at 409.70 *μ*mol/L in critical ones, which was in accordance with the serum VEGF levels in each type of severity group. 

## 4. Discussion

Using large cohort of serum samples from HFRS patients, we showed that the level of serum VEGF was dramatically elevated in HFRS patients after HTNV infection. The dynamic analysis showed that the elevation began from the onset of fever then gradually increased, but reached the peak level at different stages according to the clinical severity of the disease, suggesting that VEGF was indeed involved and may play certain role in the course of the disease.

Clinical and pathological findings showed that Hantavirus antigens were prominently presented in capillary endothelial cells of the pulmonary or kidney. The pathogenic Hantaviruses specifically target endothelial cells with the *β*3 integrin as the receptor for infection. In the kidney, the major target organ of HTNV infection, VEGF, and its receptors are widely expressed in different types of cells. VEGF is expressed most prominently in glomerular podocytes, distal tubules, and collecting ducts, whereas VEGF receptors are mainly expressed by endothelial cells of glomerular and peritubular capillaries [33, 34]. The constitutive expression of VEGF in the glomerular epithelial cells essentially maintains normal glomerular functions and provides a filtration barrier [11, 20]. In fact, VEGF binding to VEGFR2 on endothelial cells could result in phosphorylation of the receptors to transduce the major signals for angiogenesis [35, 36]. However, the Hantaviruses infection could block the function of the complex of VEGFR2 and *β*3 integrin, which may contribute to cytoskeletal reorganization in an HTNV-induced hyperpermeability response to VEGF [37, 38]. Endothelial cell monolayers are not permeabilized by Hantavirus infection alone, and pathogenic Hantaviruses direct endothelium hyperpermeability by sensitizing endothelial cells hyperresponsive to VEGF, or indirectly through the induction of nitric oxide and prostacyclin, and this alters the fluid barrier function of endothelial cell adherence junctions, resulting in enhanced paracellular permeability [39–43]. Thus, the high level of serum VEGF at oliguric stage of HFRS patients may be involved in the pathogenesis of renal injury with the manifestation of generalized capillary damage and broadened edema, which was in accordance with the high value of sCr generally at oliguric stage.

Since pathogenic Hantaviruses could use *β*3 integrins on the surface of endothelial cells and platelets for attachment and induce the adherence of quiescent platelets to the endothelial cells surface [44, 45], the Hantaviruses could interact with platelets and endothelial cells at the same time after infection, and quiescent platelets would probably form a covering layer on the surface of endothelial cells and dramatically change the adherence properties of the endothelium [11, 46, 47]. It has been reported that the platelets covering endothelial cells within the pulmonary microvasculature might alter oxygen exchange and contribute to hypoxia and hypoxia-inducible factor-1 *α*-(HIF-1 *α*-) directed VEGF induction, which, in turn, causes pulmonary edema [48–50]. Since the glomerular podocytes and many immune cells could also secrete VEGF, the hypoxia-induced endothelial cells may be one of the sources secreting VEGF that could explain for our observation that the serum VEGF level was elevated in HFRS patients.

The most obvious difference of the serum VEGF level was performed during convalescence stage of the patients, at which the serum VEGF level was higher in patients with more severe outcome of HFRS. Since the viral load of HTNV was undetectable and the disease was almost recovered at convalescence stage [51], we speculated that the high level of VEGF at convalescence after the clearance of HTNV may play its normal physiological function to promote the recovery process of impaired vascular endothelial cells and reverse the renal dysfunction to some extent. Therefore, only the mild patients with little renal injury had the normal level of VEGF at convalescence, whereas the sustained highest level of serum VEGF was observed in the critical patients, who needed more VEGF to self-repair for the serious renal capillary damage. With consistency of the sCr concentration at convalescent stage of the patients, the higher sCr level was kept at convalescence and the higher VEGF level was sustained. 

The dynamic changes of serum VEGF level showed that the peak level of serum VEGF in mild patients was performed at oliguric stage and the VEGF levels during febrile/hypotensive to oliguric stages were higher than the other three groups. As we reported previously, the milder HFRS usually had lower viral load after HTNV infection [51]. The higher level of serum VEGF but lower viral load during the acute stage of mild HFRS compared with other groups indicated that the viral load might be a more important factor influencing the severity of the renal endothelial injury. However, the mechanisms by which the patients with high level of serum VEGF and low viral load at acute stage could lead to mild severity of HFRS are needed to be investigated further.

## 5. Conclusion

In summary, the dynamic changes of the elevated serum VEGF after HTNV infection correlated with different severities of HFRS. The high level of serum VEGF at the oliguric stage in HFRS patients may be involved in the pathogenesis of renal injury. However, the sustained high level of serum VEGF at diuretic or convalescence stage would probably contribute to the renal recovery after the clearance of the virus. The different effects of serum VEGF during the course of HFRS may help us to better understand the mechanism of the renal pathological damage and the renal self-repair after Hantavirus infection. Although the VEGF-endothelial cell responses to the pathogenesis of HFRS have been reported in many studies, the function of VEGF as a recovery factor during the convalescence after HTNV infection is still needed to be further investigated. 

## Figures and Tables

**Table 1:** Characteristics of the HFRS patients with different severities.

HFRS severity	Patient number	Male (%)	Mean age (years)	Mean level of maximum blood urea (mmol/L)^a^	Mean level of maximum serum creatinine (*μ*mol/L)^a^	Mean level of maximum leukocyte (*1000/*μ*L)^a^	Mean level of nadir platelet (*1000/*μ*L)^a^	Mean level of nadir serum albumin (g/L)^a^	Mean level of maximum atypical lymphocyte (%)^b^	Mean level of serum maximum VEGF (pg/mL)^c^	Mean level of serum VEGF at convalescence (pg/mL)
Mild	13	69.2	42.1 (29.8–54.5)	11.8 (6.5–17.2)	194.6 (101.6–287.5)	15.2 (11.1–19.3)	54.8 (35.0–74.6)	34.2 (31.4–37.0)	12.4 (7.6–17.3)	564.9 (487.9–641.9)	193.3 (67.9–454.5)
Moderate	21	71.4	42.9 (34.8–51.1)	15.2 (10.5–20.0)	245.6 (158.6–332.6)	19.6 (13.5–25.7)	44.4 (29.7–59.1)	30.4 (28.3–32.5)	10.4 (8.5–12.3)	603.1 (369.9–836.3)	429.8 (198.2–661.5)
Severe	26	53.8	40.7 (32.9–48.5)	21.7 (16.1–27.3)	403.5 (250.4–556.7)	20.0 (14.5–25.4)	21.1 (10.7–31.6)	28.3 (26.0–30.7)	11.3 (8.3–14.3)	603.8 (398.0–809.5)	494.3 (398.8–589.9)
Critical	28	60.7	46.4 (38.5–54.2)	32.2 (27.4–37.1)	637.7 (544.6–730.8)	35.5 (21.5–49.6)	14.2 (8.3–20.1)	24.0 (21.4–26.5)	12.5 (9.6–15.4)	612.5 (516.4–708.5)	765.7 (429.3–1102.2)*

^a^The clinical parameters were statistically analyzed with the maximum or minimal records of each parameter during the course of the disease. The data were presented as mean (95% confidence interval).

^b^The atypical lymphocyte morphology was defined as either prolymphocytes or lymphocytes with cleaved nuclei or lymphoplasmacytoid cells by microscope. The data were presented as mean (95% confidence interval).

^c^VEGF: vascular endothelial growth factor. The level of serum VEGF was statistically analyzed with the maximum value of each patient during the hospitalization. There was no difference between the four different severity groups, *P* > 0.05.

*There was significant difference of serum VEGF level between the critical group and other group (mild, moderate, or severe) at convalescence stage of the disease, *P* < 0.05.

**Table 2:** Characteristics of the HFRS patients at different stages of the disease.

HFRS stage	Sample number	Mean level of blood urea (mmol/L)^a^	Mean level of serum creatinine (*μ*mol/L)^a^	Mean level of leukocyte (*1000/*μ*L)^a^	Mean level of platelet (*1000/*μ*L)^a^	Mean level of serum albumin (g/L)^a^	Proteinuria^b^	Mean level of serum VEGF (pg/mL)^c^
Febrile	32	14.5 (8.7–20.3)	182.5 (133.3–231.8)	17.1 (9.0–25.2)	53.9 (40.4–67.4)	30.7 (28.8–32.7)	(−)–(+)	454.9 (351.9–557.9)
Hypotensive	15	16.3 (11.8–20.8)	250.2 (170.5–329.9)	28.9 (18.8–39.1)	25.4 (15.6–35.1)	25.6 (22.8–28.3)	(±)–(+++)	473.7 (312.4–634.9)
Oliguric	34	18.6 (15.5–21.8)	391.3 (312.2–470.5)	14.5 (11.1–17.9)	95.1 (62.5–127.6)	30.1 (28.3–31.9)	(+)–(+++)	596.0 (460.5–731.4)
Diuretic	54	10.1 (8.5–11.8)	243.9 (195.0–292.9)	7.7 (7.0–8.5)	199.0 (175.4–222.7)	37.0 (35.7–38.4)	(±)–(++)	752.1 (620.2–884.1)*
Convalescence	25	8.9 (5.1–12.7)	150.2 (102.1–198.2)	6.3 (5.5–7.1)	227.9 (161.4–294.5)	39.9 (37.4–42.5)	(−)–(+)	543.0 (405.0–681.0)
8 months after	37	—	—	—	—	—	—	201.2 (161.7–240.7)**

^a^The clinical parameters were statistically analyzed with the records on the same day that we had obtained and detected the VEGF from the serum samples. The data were presented as mean (95% confidence interval).

^b^The severity range of proteinuria of the patients at different stages of HFRS.

^c^VEGF: vascular endothelial growth factor. The mean level of serum VEGF was statistically analyzed at each stage without considering the different severity of the disease.

*The serum VEGF level at diuretic stage of the patients was higher than that at other stages, *P* < 0.05.

**The serum VEGF level of the patients 8 months after discharge was significant lower than that at any stage of the hospitalization, *P* < 0.01.
